# Comparing the effectiveness of metformin with lifestyle modification for the primary prevention of type II diabetes: a systematic review and meta-analysis

**DOI:** 10.1186/s12902-023-01445-9

**Published:** 2023-09-18

**Authors:** Sayedah Sarah Mousavi, Seyedeh Mahdieh Namayandeh, Hossein Fallahzadeh, Masoud Rahmanian, Mehdi Mollahosseini

**Affiliations:** 1 Department of Biostatistics and Epidemiology, School of Public Health, Shahid Sadoughi University of Medical Sciences and Health Services, Shohaday-E-Gomnam Blvd., Alem Sq., Yazd, Iran; 2 Center for Healthcare Data Modeling, Departments of Biostatistics and Epidemiology, School of Public Health, Shahid Sadoughi University of Medical Sciences and Health Services, Shohaday-E-Gomnam Blvd., Alem Sq., Yazd, Iran; 3 Yazd Diabetes Research Center, Shahid Sadoughi University of Medical Sciences and Health Services, Shohaday-E-Gomnam Blvd., Alem Sq., Yazd, Iran; 4 Department of Community Nutrition, School of Public Health, Shahid Sadoughi University of Medical Sciences and Health Services, Shohaday-E-Gomnam Blvd., Alem Sq., Yazd, Iran

**Keywords:** Diabetes, Lifestyle modification, Metformin, Primary prevention, Systematic review, Meta-analysis

## Abstract

**Background:**

Concerning ascending trend in the prevalence of chronic type II diabetes, prevention and the development of an effective approach after the recognition of at-risk individuals is crucial. This study aims to investigate comparing the influence of lifestyle modification and metformin interventions in the prevention of type II diabetes developments.

**Method:**

The search was conducted using PubMed, Google Scholar, Scopus and Web of Science databases. The inclusion criteria include randomized controlled trials (RCT) which studied both lifestyle modification and metformin interventions in the population above 18 years old without a history of any type of diabetes. After excluding studies with intervention time of fewer than 6 months, a systematic review and meta-analysis were performed to evaluate relative risk (RR) with a confidence interval (CI) of 95% of type II diabetes development.

**Results:**

Data from 5 studies were included in the meta-analysis. The population also consists of individuals with a mean age of 50 years old with BMI and FBS of 35.5 and 104.7 mg/dl respectively. Participants range of prevention years was between 2–3 years with a mean of 2.8 years. Lifestyle modification decreases the probability of the incidence of type II diabetes by 25.3% (RR: 0.747, 95% CI, 0.6—0.92) compared to the metformin intervention (*p*-value = 0.007). Our results indicate that long-term lifestyle modifications can prevent diabetes type II and decrease diabetes mellitus incidence down to one-quarter in comparison to metformin.

**Conclusion:**

Lifestyle modification can be more efficacious than metformin in diminishing the incidence of type II diabetes. Therefore, lifestyle modification can be a therapeutic strategy for controlling type II diabetes incidence, especially in high-risk individuals.

## Key messages

This meta-analysis includes randomized clinical trials that directly evaluated two lifestyle modification and metformin treatment interventions. Our results indicate that long-term lifestyle modifications can prevent diabetes type II and decrease diabetes mellitus incidence down to one-quarter in comparison to metformin.

## Introduction

The world health organization (WHO) warning about the worldwide ascending trend of diabetes prevalence took place for the first time in 1980 with the report of 108 million of affected people [1]. It is predicted that 463 million people which equals to 9.3% of the adult population (20–79 years old) would develop diabetes by 2019 [2]. It is expected that this number reaches 578 million people by 2030 and to700 million affected people by 2045 which equals to 10.9% of the population [2]. The number of affected people in Iran are estimated to be about 5 million people (3.9–6.6) and the number will be doubled by 2040 [2]. Approximately 90% of all diabetics are affected by the type II diabetes [2]. Even after four decades, diabetes is still among the 10 main leading reasons of mortality while half of these deaths happen in people under 60 years old [3]. The growing increase in incidence of the diabetes in adolescents to type II diabetes in recent years along with their increased survival rate resulted in the rise in the total prevalence of the disease [4]. Given that the incidence is a merit for the estimation of risk and is independent of time and people’s survival, therefore, is a precious merit for evaluating the preventative actions in the population [5]. A complicated collection of genetic, demographical, and environmental factors have led to an increase in the number of people with type II diabetes including weight gain and a decrease in exercise, probably due to the civilization and ageing of the population [3]. It is obvious that the incidence and prevalence of diabetes continue to grow in both developing and developed countries, but a decrease in the age of incidence doubles the importance of the matter [6]. Moreover, the chronic nature of diabetes and its consequences intensify the pain for the patient and his/her family which is costly for the health system and society as well [2].

Pre-diabetic individuals are more likely to develop diabetes in future which makes it possible to predict new diabetic cases [7]. Therefore, seeking the primary prevention, diagnosis and management of pre-diabetic cases would be a health priority and more efficacious in controlling diabetes incidence [7]. Therefore, in line with the diabetes prevention and control program, identifying people at risk and preventative interventions are necessary and cost-effective. Therefore, we aims to find the best approach to prevent type II diabetes by reviewing randomized clinical trial (RCT) studies. After identifying people at risk, purposeful planning is needed to intervene and select which one of the two approaches of metformin prescription and lifestyle modification. In addition, the investigation of these interventions in the long term makes this study different from other studies. The most effective and efficient intervention can greatly help advanceour goals and lead to fundamental and comprehensive decisions in the country's health care system.

## Methods

### Protocol registration

We registered the protocol for this systematic review with PROSPERO (CRD42021237135). The protocol article was published in the CCB journal [8].

### Data sources and searches

This study is a systematic review and meta-analysis of RCT studies. The aim of this study is to answer the question that which intervention (metformin administration or lifestyle modification) is more effective in the prevention of type II diabetes. First, a searching strategy specifically in each of the PubMed, Google Scholar, Scopus, Cochran Library, and Web of Science data bases to find relevant papers with a combination of keywords including diabetes, primary prevention, lifestyle, and metformin was designed and performed without any language or time restrictions. Structured search terms are words related to the subject and topics of the medical subject (MESH). The search strategy and selection criteria for studies are based on population, intervention, comparison and outcome (PICO) [9]. The population is all individuals over 18 years old. Intervention groups include two lifestyle modification interventions based on lifestyle change therapy (TLC) or diabetes prevention program (DPP), and metformin drug intervention.

### Study selection

Then we directly compared the participants in these two groups. The primary outcome of this study is the incidence of type II diabetes. After trying different strategies, finally, the search strategy with the least filter was used and the least restriction was selected. The search was performed separately by two project partners in the mentioned databases with the specified search strategy.

After preparing the initial list of articles, the titles get observed and duplicates were removed. Then, the titles and abstracts of the remaining articles were carefully studied. Inclusion criteria include RCT studies that evaluated two lifestyle modifications and metformin interventions on a population over 18 years old for primary prevention of type II diabetes. The full texts of the articles get reviewed and finally, the qualified articles were selected. Any disagreement in this regard was resolved through discussion with the third researcher. EndNote software version X8.0.1 was also used for systematic reviewing.

### Data extraction and quality assessment

The quality of RCT studies was evaluated according to Jadad criteria (Table 1). Then the eligible articles and information about the author's name, year of publication, source, age and sex of participants, place of study, type of study, sample size, duration of follow-up of participants, more detailed information on lifestyle modification and metformin interventions, as well as information on the outcome of type II diabetes, were included in the Excel file, which is summarized in Table 2.

**Table 1:** Quality scoring of studies based on the Jadad scale

Study	Randomization	Blinding	An account of all patients	Total Jadad score
DPP [10] (2002)	1	0	1	2
IDPP-1 [11] (2006)	1	0	1	2
Lu, Y. H. et al. [12] (2011)	1	0	1	2
D-CLIP [13] (2016)	2	0	1	3
PREVENT-DM [14] (2017)	2	2	1	5
Andreadis, E. A. et al. [15] (2009)	0	0	0	0

**Table 2:** Type of interventions and inclusion criteria

The study, Author (publish year)	Follow up (year)	Intervention	Control	Diagnostic criteria	Subject type	Evaluated outcome
DPP, Knowler, W. C [10] (2002)	2.8	The lifestyle modification program contained 16 individual counselling sessions on diet, exercise, and behaviour modification over 24 weeks. Goals were 7% weight loss and ≥ 150 min physical activity per week. Maintenance: subsequent individual sessions (usually monthly) and group sessions (the Diabetes Prevention Program)	The Metformin group received 850 mg twice daily	ADA 1997	Perdiabetes with IGT and IFG	Diabetes incidence, weight, BMI, FPG
IDPP1, Ramachandran, A [11]. (2006)	3	The lifestyle modification group encouraged to exercise 30 min/per day and improve their diet during monthly phone calls and in person every six months	The Metformin group received 250 mg of Metformin twice daily	WHO 1999	Prediabetes with IGT	Diabetes incidence
D-CLIP, Weber, M. B [13] (2016)	2.54	Weekly classes included 16 core intervention classes on active lifestyle changes in months 0–4, followed by 8 maintenance classes in months 5–6. participants had two study goals: 7% weight loss and 150 min weekly of moderate-intensity exercise Plus metformin at a dose of 500 mg twice daily	A single day with one-on-one visits with a physician, a dietitian, and a fitness trainer and one group class on diabetes prevention	ADA 2003	overweight or obese prediabetes with iIGT or iIFG or IGT & IFG	Diabetes incidence
Lu, Y. H. et al. [12] (2011)	2	Intervention participants received lifestyle coaching in person every 3 months and by phone monthly and those with IFG received metformin 250 mg 3 times daily	Received basic education and usual care	ADA 2003	Prediabetes with IGT or IFG	Diabetes incidence
PREVENT-DM, O'Brien, M. J [14]. (2016)	1	Intensive lifestyle interventions (ILI) were adapted from the Diabetes Prevention Program’s ILI and delivered by community health workers (promotoras) over 24 sessions	The Metformin group received metformin 850 mg twice daily	impaired fasting glucose (fasting plasma glucose of 100–125 mg/dl), elevated haemoglobin A_1_c (HbA_1_c) of 5.7%–6.4%	Latinas prediabetes	Diabetes incidence, weight, BMI, FPG, HbA_1_c

*ADA* American Diabetes Association Statements, *IGT* Impaired Glucose Tolerance, *IFG* Impaired Fasting Glucose, *iIGT* Isolated impaired glucose tolerance, *iIFG* Isolated impaired fasting glucose, *D-CLIP* The Diabetes Community Lifestyle Improvement Program, *IDDP* Indian diabetes prevention program, *PREVENT-DM* The Promotora Effectiveness Versus Metformin Trial

### Statistical analysis

Data were extracted to compare the effect of these two intervention methods. studies with proper quality and follow-up time of more than 6 months were selected and numbers related to the effect size of type II diabetes mellitus (SD, RR) were extracted with a confidence interval of 95%. Finally, a stochastic effects model was used to evaluate pooled effect size. To calculate, the incidence rate was considered as one when the incidence rate was zero for both intervention groups. Chi-square and I^2^-Index tests were used to investigate the statistical heterogeneity among the studies presented in the meta-analysis. The I^2^ above 25 and *P*-value greater than 0.1 means heterogeneity [16]. Emission bias was evaluated visually using a funnel diagram and egger test, and trim and fill method. Data analysis was performed using Comprehensive meta-analysis software version 2.2064.

## Results

### Characterization of studies

**Fig. 1:**
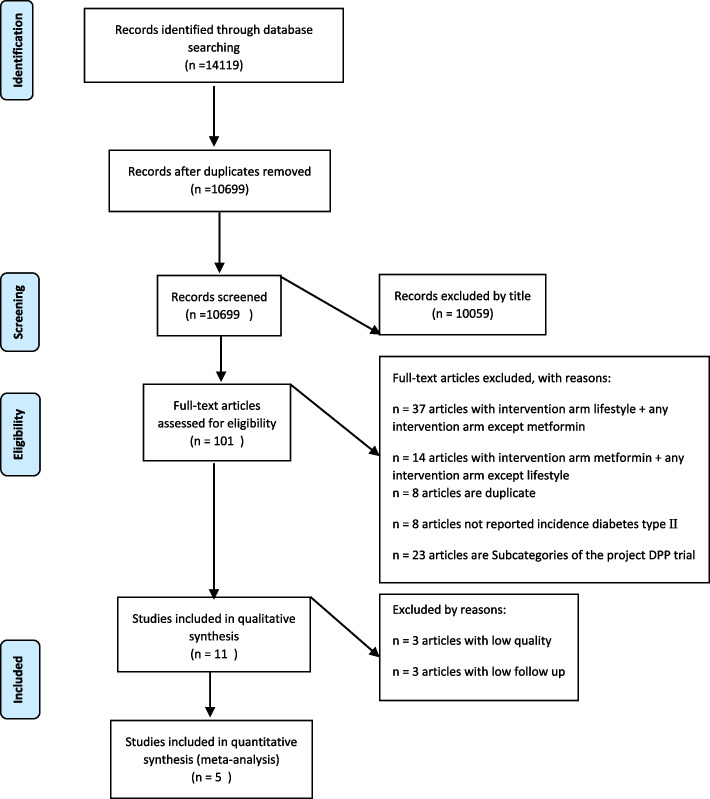
Flow diagram of the study screening and selection process by PRISMA guidelines 2009. DPP: diabetes prevention program

The total number of articles searched in the three databases PubMed, Scopus and Web of science were 14,119 articles. Also, ensuring a comprehensive search of articles, the first 300 articles searched in Google Scholar was read. Then 2789 duplicates were removed using EndNote software and another 631 articles were added and deleted to these duplicates during reading the titles of the articles. Given that the selection criteria for the article included in our study include RCT comparing lifestyle modification intervention with metformin in the population over 18 years of age to evaluate the outcome of type II diabetes. 10,059 articles were excluded via reading the title and the abstract of 640 articles was reviewed. Of these, 41 abstracts were not available and 498 articles did not meet the inclusion criteria. 101 articles were selected to read in full text. After discarding studies that examined only one of two lifestyle modification interventions, or metformin, 50 articles were finally included in the meta-analysis. Of these, 23 articles were subsets of the US Diabetes Prevention Program (DPP) pilot study project, all of which were considered as one article. Three articles did not report the incidence of diabetes, but despite correspondence to obtain the number of cases of diabetes in the intervention groups, no response was received, and also the initial outcome reviewed in 5 articles was non-type II diabetes and were excluded. Three articles were deleted due to the low quality of the trial (score based on jaded < 2) and three articles were deleted due to the short follow-up time (< 6 months) finally data from 5 articles were entered into a meta-analysis to investigate the outcome of type II diabetes (Fig. 1).

The total population of the five trials included in the meta-analysis with the outcome of diabetes was 3143 people. 42% of the participants were men. The baseline population profile with a mean age of 50 years, and BMI, and FBS were 35.2 and 104.7 mg/dl, respectively. Also, the range of follow-up years of participants is 2 to 3 with an average of 2.8 years. Table 3 shows the demographic characteristics of these five trials.

**Table 3:** Characteristics of studies included in the systematic review and meta-analysis

Author Study	Year of start study	Primary outcome	Country	Follow-up (year)	Sample size	Male baseline	Age baseline Mean ± SD	BMI Baseline Mean ± SD	FPG Baseline Mean ± SD	BMI After intervention MD ± SD	FPG After intervention MD ± SD
Knowler, W**.** C. [10] DPP	1999	Diabetes incidence	USA	2.8	2152	708	50.6 ± 10.7	34 ± 6.7	106.5 ± 8.3	- 1.45 ± 1.96	- 0.76 ± 11.84
Ramachandran, A. [11] IDPP1	2002	Diabetes incidence	India	3	266	211	45.9 ± 5.8	25.8 ± 3.5	97 ± 0.75	Not reported	Not reported
Weber, M B. [13] D-CLIP	2012	Diabetes incidence	India	2.54	576	364	44.4 ± 9.3	27.9 ± 3.7	102.3 ± 0.5	Not reported	Not reported
Lu, Y. H. [12]	2005	Diabetes incidence	China	2	181	95	63.5 ± 6.9	27 ± 3.5	106.9 ± 0.47	26.67 ± 3.66	5.99 ± 0.88
O'Brien, M. J. [14] PREVEN T-DM		Weight	USA	1	62	0	45.6 ± 12	33.8 ± 6.9	96.1 ± 8.8	- 1.2	0.83

*SD* Standard deviation

### Comparison of the efficacy of two lifestyle modification and metformin interventions

**Fig. 2:**
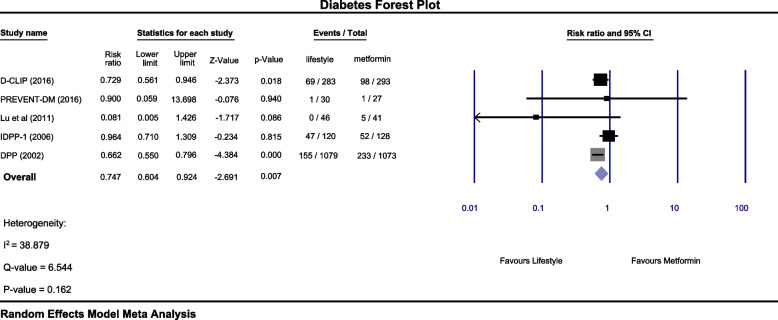
Forest plot comparing the effect of lifestyle modification interventions with metformin on the incidence of type II diabetes. Relative risk estimation (RR) with confidence interval (CI) 95%. Each square shows the relative effect size and the horizontal line of the 95% confidence interval in each study. The area of each square is proportional to the relative weight of the study. The diamond shows the estimated size of the overall effect. The Diabetes Community Lifestyle Improvement Program (D-CLIP), The Promotora Effectiveness Versus Metformin Trial (PREVENT-DM), Indian diabetes prevention program (IDDP), Diabetes prevention program (DPP)

The incidence of type II diabetes was estimated to be 8.5 cases per 100 person-years in the lifestyle modification intervention group and 9.9 cases per 100 person-years in the metformin intervention group. Also, the relative risk assessment of RCT studies showed that lifestyle modification intervention 25.3 times (RR 0.747: 95% CI: 0.6 – 0.92) reduced the risk of type II diabetes compared to metformin (*p*-value = 0.007). The heterogeneity rate was 39% I^2^ and Cochran test was estimated to be 6.5 (*p*-value = 0.16) (Fig. 2).

**Fig. 3:**
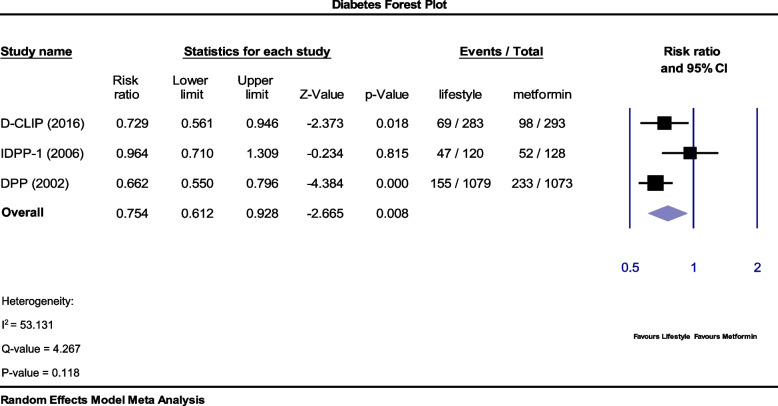
Forest plot comparing the effect of lifestyle modification interventions with metformin on the incidence of type II diabetes. Relative risk estimation (RR) with confidence interval (CI) 95% and eliminate relatively low-weight trials

Two trials, PREVENT-DM and Lu et al., had a much lower weight average than the other studies due to their small sample size, and there was little change in relative risk when excluded from analysis (RR = 0.754); But the degree of inconsistency increased slightly (53.1% = I2). Therefore, our meta-analysis is not affected by these two trials (Fig. 3).

### Publication bias

**Fig. 4:**
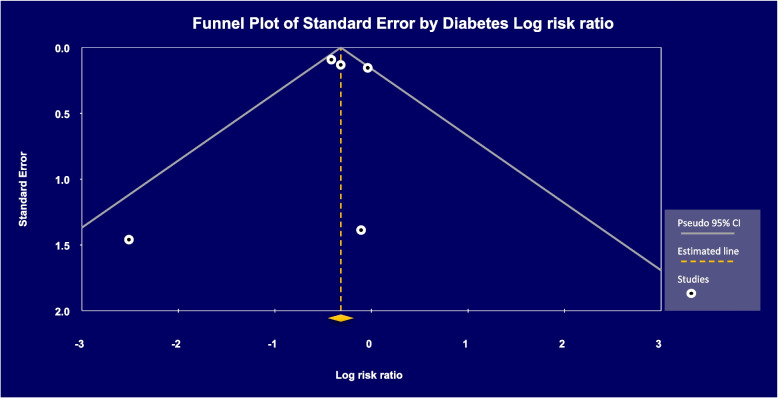
Type II diabetes. The funnel diagram is symmetric. Number of studies = 5, egger regression interpretations: t-value = 0.27642, df = 3, *p*-value = 0.80017

A funnel diagram is for investigation of the diffusion bias. In this diagram, each point represents a study entered into the meta-analysis, drawn according to their effect (x-axis) and standard error (y-axis). In the evaluation of diffusion bias for studies with the outcome of type II diabetes, the funnel diagram visually showed symmetry (Fig. 4) and the non-significance of the Egger test also indicates the absence of diffusion bias (*p*-value = 0.80017). Also, no studies were hypothetically added to the chart by performing the trim and fill test; this means that small or ineffective studies may have been included in our meta-analysis.

## Discuss

In this systematic review and meta-analysis, we investigated two lifestyle modification interventions and the prescription of metformin to prevent type II diabetes and the effect of these interventions on the incidence of diabetes. This meta-analysis includes RCT studies that directly evaluated two lifestyle modification and metformin treatment interventions. The results indicate the effectiveness of lifestyle modification compared to metformin for the prevention of diabetes. According to the relative risk of 0.75 (95% CI: 0.60–0.92) obtained in the time range of 6 months to 3 years, the incidence of diabetes has decreased by 25% in the lifestyle modification intervention compared to metformin. Both lifestyle modification and drug therapy are effective in preventing diabetes compared to the control group. It is estimated that the combination of dietary methods and physical activity in lifestyle modification intervention is more effective in reducing the risk of diabetes and has a synergistic effect. Also, in the discussion of drug interventions, weight-reducing drugs such as glitazone and metformin reduce the risk of diabetes in high-risk people more than other drugs through insulin sensitizers [17]. For example, lifestyle modification intervention with a relative risk of 0.72 (95% CI: 0.86–0.60) compared to drug treatment with a relative risk of 0.95 (1.14–0.79: 95% CI) in the follow-up after stopping the intervention for a long time in the prevention of diabetes. has been more effective and shows that the protective effect of lifestyle modification is greater in the long term, which can be the reason for its superiority compared to metformin [17, 18]. The effects of drug interventions are limited to the time of their use and do not permanently change the basic pathophysiology of insulin resistance or β-cell dysfunction [17]. When we compare the results of the DPP clinical trial with a follow-up of 15 years with the results of the same trial in the initial interventions with a follow-up of 2.8 years, we find that both lifestyle change interventions and metformin were more effective in reducing the risk of diabetes than the control group [10, 19]. However, by conducting a meta-analysis on trial studies, they found that among the participants who received the drug intervention and who received the placebo, when comparing the follow-up period of active intervention and follow-up after cessation of intervention, the relative risk of diabetes after cessation of intervention was not significant. While this trend shows a reduction in the risk of diabetes for lifestyle intervention in both follow-up periods, the lifestyle modification approach seems more useful in preventing diabetes in the long run. While this trend shows a reduction in the risk of diabetes for lifestyle intervention in both follow-up periods, the lifestyle modification approach seems more useful in preventing diabetes in the long run [17]. However, in general, any intervention for people at risk can be effective and reduce the risk of type 2 diabetes. The study of two risk factors related to diabetes such as obesity and reduced physical activity has shown that lifestyle interventions aimed at improving these two factors have been more effective in reducing the incidence of diabetes. It also seems that the higher the BMI of individuals, the more effective the lifestyle intervention. Although drug interventions also played a role in reducing the incidence of diabetes, the effects were not stable after stopping the treatment, so the incidence of diabetes increased after stopping the use of two drugs, troglitazone and metformin [20]. Although this study did not directly evaluate the two lifestyle modification and metformin interventions, the final results of our meta-analysis confirm and agree with these findings. On the other hand, a network meta-analysis (NMA) study shows that lifestyle modification intervention has performed better compared to 12 other therapeutic interventions for the prevention of type II diabetes in high-risk populations. However, in an indirect comparison between lifestyle modification and metformin, the odds ratio of diabetes showed a 14% reduction, but it was not statistically significant (95% CI: 1.25–0.6, OR: 86%) [21]. This inconsistency with our study is probably due to the smaller number of included studies.

### Advantages and limitations

Despite that our meta-analysis directly compared the two lifestyle modification and metformin interventions with a timeless and linguistic search strategy, the severity of heterogeneity between RCT studies was low and the meta-analysis of diabetes incidence was not affected by diffusion bias. However, we only analyzed randomized trials with a follow-up period of more than 6 months. Meanwhile, we only analyzed randomized trials with a follow-up period of more than 6 months. The lack of follow-up of participants, in the long run, is another limitation of the study that can change our results because people's acceptance of lifestyle changes or long-term drug intervention has not been measured. Furthermore, this meta-analysis did not evaluate surgical interventions or other drugs.

## Conclusion

Due to the increasing trend of diabetes and the chronic nature of the disease, primary prevention or prevention of the first type is necessary and cost-effective. Therefore, we decided to answer the research question of whether lifestyle changes are more beneficial in reducing long-term diabetes or whether treatment with metformin might be a better solution. Our results revealed that after diagnosing a person as pre-diabetic, a lifestyle modification approach for more than 6 months can be effective in reducing the incidence of type II diabetes and reducing the incidence of diabetes by 25% compared to metformin. Depending on the individual’s condition and level of acceptance, each of these two interventions can be applied together or alone. Though, concerning the cultural resources and contexts, we suggest that preventative diabetes incidence plans be based on lifestyle changes at the community level.

## Data Availability

The data that support the finding of this study are available from the corresponding author upon reasonable request.
